# Economic Benefits of Reduced Waiting Times for Elective Surgeries: A Systematic Literature Review

**DOI:** 10.7759/cureus.79417

**Published:** 2025-02-21

**Authors:** Rok Hren, Nada Abaza, Baher Elezbawy, Ahmed Khalifa, Ahmad N Fasseeh, Naeema Al Gasseer, Zoltán Kaló

**Affiliations:** 1 Evidence Synthesis, Syreon Research Institute, Budapest, HUN; 2 Health Economics, Syreon Middle East, Cairo, EGY; 3 Evidence Synthesis, Syreon Middle East, Alexandria, EGY; 4 Health Economics, World Health Organization, Cairo, EGY; 5 Modelling, Syreon Middle East, Alexandria, EGY; 6 WHO Regional Office for the Eastern Mediterranean (EMRO), World Health Organization, Cairo, EGY; 7 Health Economics, Center for Health Technology Assessment, Semmelweis University, Budapest, HUN

**Keywords:** economic evaluation, elective surgery, systematic review, waiting list, waiting times

## Abstract

This systematic literature review aims to explore studies that assess the cost-effectiveness of reducing waiting times in elective surgeries. We conducted a systematic search of the MEDLINE/PubMed and Embase/Scopus electronic databases on March 12, 2024. Eligibility criteria included elective surgery and economic evaluation of waiting times, while transplantation surgery procedures were excluded. Due to the anticipated heterogeneity of the studies, the review was presented in a narrative synthesis format. Nine articles met the inclusion criteria, covering elective surgery procedures in the musculoskeletal system (four articles), cardiovascular system (two articles), ophthalmic system (one article), and gastrointestinal tract (two articles). The evidence from this review suggests that reducing waiting times in elective surgery is highly cost-effective and often cost-saving. While it may be argued that accounting for specific elective surgeries and specific countries limits the generalizability of the findings, the review provides quantitative evidence that supports the value of reducing waiting times.

## Introduction and background

Policymakers face numerous resource limitations within healthcare systems that require careful management. For example, natural resources, such as blood and plasma, are often insufficient to meet the needs of all patients, and the production of essential technologies, like vaccines during pandemics, may not scale up quickly enough to address urgent demands. Shortages of healthcare professionals will be a growing concern in the next decades, leading to the migration of both physicians and nurses from lower-income to higher-income countries [1]. Additionally, limited infrastructure for high-cost services, such as diagnostic imaging, radiotherapy, and robotic surgery, remains a significant challenge in lower-income regions. Financial constraints pose another critical hurdle, especially for high-cost innovative medicines, with the supply of such medicines often maintained by multinational manufacturers, while affordability remains a pressing issue.

Addressing these diverse resource limitations requires targeted and strategic policy interventions. For instance, to address the shortage of healthcare professionals or the limited availability of specialist centers for elective surgical procedures, investments should focus on strengthening the most critical human resources and expanding essential infrastructure. Financial constraints can be from a purely economic perspective managed through measures such as introducing patient co-payments, which can help reduce excessive demand for healthcare services [2].

When eligible patients cannot promptly receive the necessary services or technologies, health policymakers implement waiting lists as a tool to ration healthcare and ensure equitable resource allocation [3]. However, waiting lists are only suitable for conditions where delayed access to healthcare does not lead to fatal consequences or rapid disease progression. Still, waiting for healthcare services causes discomfort, heightened pain, anxiety, and limitations in daily functioning to patients and their families [4]. Even in diseases with slow progression, waiting for long times results in poorer outcomes [5]. As a consequence, healthcare systems face ongoing pressure to minimize waiting lists [6]. Waiting times for elective surgeries are considered a key performance indicator for healthcare systems, with their reduction aimed at improving performance within healthcare facilities to deliver timely and high-quality care [7].

Waiting lists for elective surgeries and procedures remain a significant global healthcare challenge. A recent Organisation for Economic Co-operation and Development (OECD) report [8] focused on three key elective surgical procedures, including cataract surgery, hip replacement, and knee replacement, across 12 jurisdictions. For cataract surgery, median wait times exceeded 100 days in Ireland, and 200 days in Costa Rica and Slovenia, while Hungary, Spain, and Sweden reported significantly shorter median waiting times, all under 50 days. Similar disparities were observed for hip and knee replacement procedures. For hip replacement, median waiting times were below 90 days in Spain and Sweden, but notably longer in Chile (>400 days), Slovenia (>500 days), Costa Rica (>600 days), and Poland (>700 days). In the case of knee replacement, median waiting times were even longer, surpassing 600 days in Chile, Slovenia, and Costa Rica, and exceeding 900 days in Poland, with only Spain reporting waiting times below 100 days.

The issue of adverse health outcomes resulting from long waiting lists for elective surgeries is a critical policy concern, and previous efforts to address this challenge have often centered around increases in funding, yielding inconsistent results [9]. Public hospitals frequently encounter limitations in their capacity to improve performance due to resource constraints, as the supply of surgical services struggles to match the rising demand [10]. The COVID-19 pandemic is a poignant example, prompting healthcare institutions to either navigate the necessity of expediting patient discharges or making significant adjustments in surgical priorities, resulting in even indefinite postponement of certain elective surgeries [11]. Recent research conducted by Quercioli et al. [12] proposed that successful strategies to reduce waiting times for elective surgeries should adopt a multidimensional approach, focusing on improvements in waiting list management, surgical scheduling, surgical pathways, and the optimization of operating room utilization. Moreover, findings by Rathnayake et al. [13] have indicated the importance of employing explicit prioritization tools equipped with a standardized scoring system based on evidence-based criteria.

The economic evaluation conducted by Koopmanschap et al. [14] presented various scenarios of waiting times and their potential impacts on health outcomes, costs, and cost-effectiveness within healthcare systems. This theoretical study emphasized that the influence of waiting time on cost-effectiveness is highly dependent on specific circumstances, with implications ranging from minimal to significant, particularly when health deterioration during the wait is irreversible.

Given that rate limiting healthcare resources to induce waiting time in elective surgeries can be alleviated by significant financial investment and capacity development, it is crucial to understand the economic value of such policy initiatives. As a starting point, we conducted a systematic literature review with the intention of focusing exclusively on studies that have conducted economic evaluations in the clinical setting. Considering the persistence of prolonged waiting times for elective surgeries, this review of economic evaluations has the potential to provide valuable insights for both healthcare policymakers and healthcare professionals in different jurisdictions.

The results of this study were previously presented as a conference abstract at the ISPOR Europe Conference in December 2024 (Code EE654).

## Review

On March 12, 2024, a literature search was conducted on the MEDLINE/PubMed and Embase/Scopus electronic databases, focusing on English language studies, without any restrictions on publication date. This systematic literature review aimed to analyze models evaluating the costs and benefits of elective surgical interventions designed to reduce waiting times, such as models comparing scenarios between surgery at the point of consultation versus placement on a waiting list. During the search process, titles and abstracts were independently reviewed by two authors (N.A. and B.E.) in a double-blinded manner using Rayyan software (Cambridge, MA) to ensure accuracy and eliminate bias; any conflicts that arose were resolved by a third author (A.N.F.). Eligibility criteria included elective surgeries and economic evaluations of waiting times, while excluding transplantation procedures due to the unique nature of waiting times associated with organ availability. Research and development studies, as well as purely methodological studies, were also excluded. Special attention was given to removing duplications, both within and across databases and studies; for example, if a study was initially published in proceedings and later in a journal, then the proceeding article was considered a non-primary publication and excluded. Studies were categorized based on the human body system under consideration in each study. Due to the anticipated heterogeneity in study designs, methodologies, outcome measures, and cost components, the review was presented in a narrative synthesis format for each study. For the same reason, a meta-analysis was not conducted, as differences in these factors reduce the reliability of pooled estimates and the accuracy of the findings.

Results

**Figure 1:**
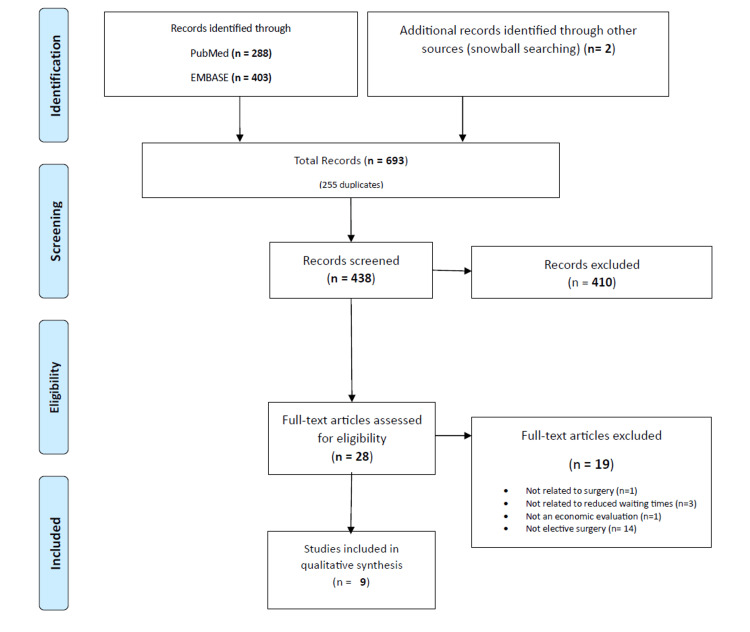
PRISMA flow diagram for selecting eligible articles in the systematic review. PRISMA: Preferred Reporting Items for Systematic Reviews and Meta-Analyses.

The article screening process is shown in the Preferred Reporting Items for Systematic Reviews and Meta-Analyses (PRISMA) flow diagram (Figure 1). In total, 288 and 403 articles were found to be of interest in the MEDLINE/PubMed and Embase/Scopus databases, respectively. After excluding duplicates and applying the exclusion criteria, first considering the title and abstract, and next, if necessary, reading the entire article, nine articles met the inclusion criteria, covering elective surgery procedures in the musculoskeletal system (four articles), cardiovascular system (two articles), ophthalmic system (one article), and gastrointestinal tract (two articles) (Table 1).

**Table 1:** Included articles reporting economic models assessing cost-effectiveness of interventions designed to reduce waiting times. GI: gastrointestinal; CEA: cost-effectiveness analysis; CUA: cost-utility analysis.

Reference	Year of publication	Country of study	Type of economic analysis (benefit measured)	Elective surgery
Musculoskeletal				
Saleh et al. [15]	1997	USA	CEA (clinical endpoints)	Total hip arthroplasty revision
Mather et al. [16]	2014	USA	CUA (utilities)	Total knee arthroplasty
Mari et al. [17]	2016	France	CUA + CEA (utilities + clinical endpoints)	Total knee arthroplasty
Karnon et al. [18]	2018	Australia	CUA + CEA (utilities + clinical endpoints)	Total knee arthroplasty
Cardiovascular				
Ribera et al. [19]	2018	Spain	CUA (utilities)	Transcatheter aortic valve implantation
Peel et al. [20]	2022	Canada	CUA + CEA (utilities + clinical endpoints)	Transcatheter aortic valve implantation
Ophthalmic				
Boyd et al. [21]	2019	New Zealand	CUA (utilities)	Cataract surgery
GI tract				
Cohen et al. [22]	2017	Brazil	CUA + CEA (utilities + clinical endpoints)	Bariatric surgery
Davis and Saunders [23]	2020	Canada	CEA (clinical endpoints)	Bariatric surgery

Methodological details of modeling approaches are presented in the Appendix. Briefly, analysis in the appendix uncovered a strong preference for Markov models, which were used in four out of nine studies, with two additional studies combining Markov and decision tree modeling. The majority (eight of nine) adopted a cohort design to enable population-level comparisons, though only two considered population-specific subgroups. The studies varied in their time horizons, with six favoring long-term projections exceeding 10 years. Sensitivity analysis was a key aspect of model robustness, with six studies using deterministic sensitivity analysis (DSA), while two incorporated both DSA and probabilistic sensitivity analysis (PSA), and one used additional sensitivity analysis techniques. A breakdown of value drivers revealed that the most commonly considered factors were (i) maintenance costs (five studies), (ii) quality of life (QoL) improvement during reduced waiting time (five studies), and (iii) mortality due to waiting (four studies).

Musculoskeletal system

A pioneering effort in assessing the economic implications of reducing waiting lists was the work of Saleh et al. [15]. They applied a cost-effectiveness analysis to the total hip arthroplasty revision (THAR), following the failure of primary hip arthroplasty. The study compared two scenarios, i.e., immediate surgery and surgery delayed by 12 months, with patients on the waiting list categorized based on their functional status (independent care or home care). The findings indicated that immediate surgery resulted in a 29.4% cost reduction ($9,254) compared to patients awaiting surgery. Sensitivity analysis further confirmed the cost-saving benefits of the immediate-surgery approach.

Mather et al. [16] evaluated the cost-effectiveness of waiting for total knee arthroplasty (TKA) in a cohort of 60-year-old individuals, and to that end, used the Markov decision model to analyze three treatment strategies: (i) immediate TKA, (ii) delayed TKA with a waiting period including a nonoperative treatment bridge, and (iii) delayed TKA with a waiting period not including a nonoperative treatment bridge. Considering a two-year waiting time, the study concluded that immediate TKA was found to be the dominant strategy (costing $1,810 less and resulting in 0.57 more quality-adjusted life years (QALYs)) compared to delayed TKA with a nonoperative treatment bridge. When comparing immediate TKA to a delayed TKA without a nonoperative treatment bridge, the resulting incremental cost-effectiveness ratio (ICER) amounted to $4,768/QALY, substantially below the US willingness-to-pay threshold of $50,000/QALY. These results included only direct costs, when indirect costs were accounted for, the incremental cost of delayed TKA either with or without a nonoperative treatment bridge became substantially higher. Sensitivity analysis showed that increasing waiting time from six months to five years increased the incremental cost of delayed TKA with a nonoperative bridge from $5,652 to $42,832 per patient when compared to immediate TKA.

Somewhat different conclusions were reached by Mari et al. [17] who compared the early TKA management strategy with the late TKA management strategy, in which the TKA surgical procedure was just one of many options. Using a Markov model over a time horizon of 30 years, the research focused on a real-world knee osteoarthritis (OA) cohort of French patients, categorized into four age groups (40-49 years, 50-59 years, 60-69 years, and 70-75 years). Both strategies, early and late TKA, encompassed a range of nonpharmacological and pharmacological treatments. Results indicated that the early TKA strategy incurred a 9.5% higher cost (€6,624) than the late TKA strategy, which was not surprising due to the higher proportion of costly surgical options (arthroscopy, osteotomy, unicompartmental knee arthroscopy, TKA) included in the early TKA strategy (29.1% patients) than in the late TKA strategy (10% patients). Specifically, TKA procedures accounted for 14.4% of patients in the early TKA strategy and only 4.1% of patients in the late TKA strategy. As the gain in QALYs of early TKA strategy over late TKA strategy was marginal (0.15 QALY over a time horizon of 30 years), the model yielded for the entire cohort an ICER of €43,631/QALY. The authors concluded that an early TKA strategy was not a highly cost-effective approach for knee OA treatment compared to the late TKA strategy.

Karnon et al. [18] explored the cost-effectiveness of implementing a private contracting model for publicly funded patients, with the goal to reduce waiting times. Using a Markov model, patients were allocated into 10 categories representing varying waiting times for TKA, ranging from six to 15 months. In standard care, it was assumed that 16% of patients would undergo TKA within six months and 17% of patients would wait over 12 months. Their analysis indicated that by employing private services to ensure that patients did not wait more than nine months for surgery, the resulting ICER would amount to A$32,831/QALY when compared to standard care, which was by authors deemed cost-effective as it was lower than ICERs associated with new pharmaceuticals that were recommended for reimbursement in Australia.

Cardiovascular system

Ribera et al. [19] assessed the cost-effectiveness of varying waiting times for transcatheter aortic valve implantation (TAVI), a cost-effective option for inoperable and high-risk patients suffering from aortic stenosis (AS). They used a Markov model to compare outcomes of immediate TAVI (with a waiting time of less than three months) to waiting times of three, six, nine, or 12 months among a cohort of 1,000 80-year-old male patients. The study revealed that while longer waiting times led to net cost-savings, they were also associated with a notable reduction in QALYs, and estimated ICERs were approximately €12,500 in savings per QALY lost. Sensitivity analysis showed that the probability of immediate TAVI being cost-effective, compared to waiting, exceeded 90% at the Spanish willingness to pay threshold of €20,000/QALY.

Peel et al. [20] examined four hypothetical strategies, including the reduction of TAVI care wait times by 10 weeks as opposed to the reference scenario with a median wait time of 19.14 weeks. Employing a probabilistic discrete-time microsimulation model with a time horizon of two years, the wait-time reduction was the primary performance indicator leading to improved clinical outcomes. The reduction in waiting times correlated with a decline in wait-list mortality (from 4.6% ± 0.7% to 2.7% ± 0.5%), fewer hospitalizations among waitlist patients (from 36% ± 2% to 21% ± 1%), and a reduced need for urgent TAVI procedures (from 18% ± 1% to 10% ± 1%). The strategy of wait-time reduction not only demonstrated increased effectiveness but also lowered costs.

Ophthalmic system

Boyd et al. [21] built upon the work of Church et al. [24], who explored the cost-effectiveness of various fall prevention strategies and found that expedited cataract surgery was a dominant strategy (costing A$160 less and resulting in 0.10 more QALYs) over the absence of surgery. Boyd et al. [21] assessed the cost-effectiveness of reducing waiting times of cataract surgery by 12 months in a population cohort aged over 65 years and found that expedited intervention resulted in ICER of NZ$10,600/QALY and was lower for the 65-69 years age group (NZ$7,000/QALY) than the oldest age group of 85-89 years (NZ14,200/QALY). The authors concluded that expedited cataract surgery appears to be highly cost-effective.

Gastrointestinal tract

Cohen et al. [22] evaluated the cost-effectiveness of various waiting times prior to bariatric surgery and immediate bariatric surgery. They used a Markov model over a time horizon of 20 years to compare immediate intervention versus waiting for one, two, four, or seven years on a cohort of patients with body mass index (BMI) > 35 kg/m2 with type II diabetes and cardiovascular disease. They found that immediate bariatric surgery was a dominant strategy compared to any delayed intervention.

Davis and Saunders [23] compared the cost of standard care with an improved bariatric care pathway with earlier sleeve gastrectomy. Employing a decision analytic model and following a cohort of 100 patients over 10 years, they found that the improved bariatric care pathway was associated with a total saving of $900,000 due to a substantial reduction in expenditures for diabetes and hypertension treatments.

Discussion

Based on this comprehensive literature review, the following inferences could be made. Waiting time for elective surgical interventions can be managed by investment into the most rate-limiting human resources or infrastructure. The opportunity cost of financial investment to reduce waiting time can be assessed by economic evaluations. Economic evaluations of reducing waiting times for elective surgery procedures, however, are rare, often lacking a systematic approach across different countries or within a single country for various elective surgery procedures. In a few published cases, the reduction of waiting times has been at least cost-effective, if not cost-saving. A study by Mari et al. [17] is an exception due to the design of the study with a higher proportion of costly surgical procedures in the cohort of patients receiving early treatment compared to those with delayed treatment.

Across different surgical types, a consistent trend emerged wherein reducing waiting times is generally cost-effective, with certain procedures demonstrating clear cost-saving benefits. In musculoskeletal surgeries, earlier interventions such as TKA and hip arthroplasty revisions were associated with reduced long-term costs and improved quality of life, with the only exception of the study of Mari et al. [17], as indicated above. Similarly, for cardiovascular procedures like TAVI, shorter waiting periods significantly lowered waitlist mortality and hospitalizations while also proving to be cost-effective. Ophthalmic and gastrointestinal surgeries followed a similar pattern, with expedited cataract and bariatric surgeries showing both health benefits and potential cost savings.

The cost-effectiveness or even the cost-saving nature of reducing waiting times not surprisingly aligns with the conceptualization of human health as a durable capital [25,26] depreciating over time and exogenously dependent solely upon an individual’s age. For this reason, hypothetical scenarios, e.g., stable health and reversible effects of waiting times [14] for elective surgeries, remain rather conjectural.

The notably limited number of publications identified through our systematic literature review indicates low interest in methodically appraising the impact of waiting times on the cost-effectiveness of both established and novel clinical interventions. In a way, this scarcity emphasizes an underexplored domain, which is somewhat unexpected given the inherent importance of conducting a thorough examination into how waiting times can influence costs and health outcomes, especially for novel surgical interventions, which substantially diverge from standard care both in terms of intervention type and associated expenditures. The recent OECD report [8] has clearly demonstrated the persistence of waiting times for elective surgeries, underlining the dire need for comprehensive studies in this domain. With the increased number of costly novel treatment options alongside an aging population, waitlists will likely worsen as countries need to deal with escalating healthcare expenditures and are compelled to prioritize patient care.

Waiting time for elective surgeries can potentially be reduced by healthcare financing approaches, such as patient copayments [4], or differential health insurance coverage among patients to reduce waiting periods [6]. Nevertheless, it is evident that both strategies raise ethical concerns, which are likely deemed unacceptable in public healthcare systems. Another unfeasible strategy would involve compromising service quality to mirror non-healthcare public services. Interestingly, none of the reviewed studies explored the cost-effectiveness of reducing waiting lists by financial approaches, because this approach may increase inequity in both healthcare financing and health provision. Complementary private health insurance and elective surgical interventions in private clinics for affluent patients may have the potential, at least theoretically, to reduce the financial constraints on publicly funded healthcare systems and may ultimately reduce the waiting lists of all patients on the waiting lists [27,28]; however, this aspect has not been addressed in the reviewed studies.

An illustrative case in point can be observed in Slovenia, renowned for one of the most advanced healthcare systems in Central and Eastern Europe, as evident from indicators, such as the infant mortality rate and the availability of reimbursed orphan drugs in the pharmaceutical market [8]. Despite these accolades, prolonged waiting times for elective surgeries, including total hip replacement, TKAs, or cataract surgeries, portray a less flattering picture of the Slovenian healthcare system among OECD countries [9]. Moreover, the considerable purchasing power of the population appears to be driving a concerning shift within the Slovenian healthcare system, particularly in the realm of elective surgeries, transitioning from a highly sophisticated social healthcare model to the most rudimentary form of out-of-pocket healthcare. Additionally, there is a potential concern regarding the adequacy of quality control measures designed to effectively monitor elective surgeries within this out-of-pocket framework. The example of Slovenia could serve as a valuable case study for other high-income and mid-income countries.

Among nine publications, eight originate from high-income countries and only one has research done in a mid-income country. This imbalance presents a limitation in the conclusions of this systematic literature review as it predominantly applies only to high-income countries. Nonetheless, the pressing issue of extended waiting times is a widely acknowledged concern, extending beyond affluent regions to the developing world [29]. This highlights the need for more economic evaluations on waiting times for elective surgeries in low and lower-middle (LMICs) regions. The World Bank's income classification criteria were used to categorize countries by income into low, lower-middle, upper-middle, and high income [30,31]. The magnitude of this challenge underscores its global policy relevance, transcending economic boundaries.

While it may be argued that accounting for specific elective surgeries limits the generalizability of our findings, the review provides evidence supporting the value of reducing waiting times. The methodological framework of our study can be readily adapted to analyze the impact of reducing waiting times in transplantation procedures, which due to unique dynamics related to organ availability differ from the primary elective surgeries analyzed in our review, such as TKAs or cataract surgeries.

## Conclusions

Long waiting lists pose a significant challenge in healthcare systems across both developed and developing countries. This review provides quantitative evidence emphasizing the importance of reducing waiting times for elective surgeries. However, the data available to evaluate the cost-effectiveness of these interventions are limited and heterogeneous, thereby rendering it necessary to develop more systematic and standardized modeling approaches.

## Appendices

Economic modeling approaches

The analysis of economic modeling approaches in the included studies unveiled a diverse range of methodologies and preferences (Table 2). Among the nine studies, a predominant reliance on Markov models was evident, with four studies employing this specific model type, and two additional studies using a combination of Markov and decision tree modeling. Eight of the nine models adopted a cohort design, allowing for population-level comparisons of early versus late surgery outcomes. However, only two models accounted for population-specific subgroups. The selection of time horizons in models varied as well, with a distinct trend in six models, which favored long-term horizons, extending beyond 10 years. Six models exclusively used DSA, while two incorporated both DSA and PSA, and one model integrated DSA, PSA, and an additional sensitivity analysis technique. These findings highlight a multifaceted approach to economic modeling within the domain of evaluation of reducing waiting times for elective surgeries. A detailed breakdown of value drivers used in the included models can be found in Table 3. The most frequently employed value drivers were maintenance costs (in five out of nine studies), improvement in quality of life during the reduced waiting time (five studies), and mortality due to waiting (four studies).

**Table 2:** Methodological details of modeling approaches in the included studies. DSA: deterministic sensitivity analysis; PSA: probabilistic sensitivity analysis. * The number of simulations in this model was 1,000,000.

Reference	Country of study	Elective surgery	Model type	Level of assessment	Population - specified subgroup	Model time horizon (years)	Societal and caregiver burden considered?	Sensitivity analysis
Musculoskeletal								
Saleh et al. [15]	USA	Total hip arthroplasty revision	Decision tree	Cohort	No	2	No	DSA
Mather et al. [16]	USA	Total knee arthroplasty	Decision tree + Markov	Cohort	No	Lifetime	Yes	PSA + DSA + other
Mari et al. [17]	France	Total knee arthroplasty	Markov	Cohort	No	30	Not mentioned	DSA
Karnon et al. [18]	Australia	Total knee arthroplasty	Decision tree + Markov	Cohort	No	15	No	DSA
Cardiovascular								
Ribera et al. [19]	Spain	Transcatheter aortic valve implantation	Markov	Cohort	Yes	Lifetime	No	PSA + DSA
Peel et al. [20]	Canada	Transcatheter aortic valve implantation	Discrete event simulation*	Individual	No	2	Not mentioned	DSA
Ophthalmic								
Boyd et al. [21]	New Zealand	Cataract surgery	Markov	Cohort	No	20	No	DSA
GI tract								
Cohen et al. [22]	Brazil	Bariatric surgery	Markov	Cohort	Yes	20	No	PSA + DSA
Davis and Saunders [23]	Canada	Bariatric surgery	Decision analytic model using power law modeling	Cohort	No	10	No	DSA

**Table 3:** Value drivers of the models in the included studies. QoL: quality of life. * No: In the context of this table, “No” denotes that the value driver was not considered or was not mentioned in the study. ^1^ Mortality due to waiting: The proportion of patients who die while waiting for an extended period, regardless of the cause. ^2^ Missed surgery due to delay: The proportion of patients who become ineligible for surgery over time, primarily due to disease progression or complications during the prolonged waiting period. ^3^ Life expectancy: Prolonged waiting times can alter patients' life expectancies. ^4^ Maintenance costs: The consistent monthly or annual expenses spent by patients for managing their disease, complications, or deterioration during the extended waiting period. ^5^ Improvement in QoL during the reduced waiting time: The reduction in waiting time leads to a notable enhancement in the quality of life experienced by patients, marked by improvements in various aspects such as physical health, emotional well-being, social interactions, and overall satisfaction with their healthcare journey. ^6^ Improvement in the long-term QoL: Improvement in patients' quality of life and prognosis over an extended duration when comparing reduced waiting times to prolonged waiting times. ^7^ Reduction in utility: Reduction in utilities or benefits experienced by patients due to the extended waiting times.

Reference	Type of elective surgery	Was the value driver considered in the model?						
		Mortality due to waiting^1^	Missed surgery due to delay^2^	Life expectancy^3^	Maintenance costs^4^	Improvement in QoL during the reduced waiting time^5^	Improvement in the long-term QoL^6^	Reduction in utility^7^
Musculoskeletal								
Saleh et al. [15]	Total hip arthroplasty revision	No*	No*	No*	Yes	No*	No*	No*
Mather et al. [16]	Total knee arthroplasty	Yes	No*	No*	Yes	Yes	Yes	Yes
Mari et al. [17]	Total knee arthroplasty	No*	No*	No*	Yes	Yes	No*	Yes
Karnon et al. [18]	Total knee arthroplasty	Yes	No*	Yes	Yes	Yes	No*	Yes
Cardiovascular								
Ribera et al. [19]	Transcatheter aortic valve implantation	Yes	No*	Yes	No*	No*	Yes	No*
Peel et al. [20]	Transcatheter aortic valve implantation	Yes	No*	No*	No*	No*	No*	No*
Ophthalmic								
Boyd et al. [21]	Cataract surgery	No*	No*	No*	No*	Yes	No*	No*
GI tract								
Cohen et al. [22]	Bariatric surgery	No*	No*	Yes	No*	Yes	No*	No*
Davis and Saunders [23]	Bariatric surgery	No*	No*	Yes	Yes	No*	No*	No*
